# Atypical Aortoesophageal Fistula with Atypical and Delayed Presentation and Negative Imaging Studies

**DOI:** 10.1155/2016/7219034

**Published:** 2016-11-14

**Authors:** Seifeldin Hakim, Mihajlo Gjeorgjievski, Lohit Garg, Molly Orosey, Tusar Desai

**Affiliations:** ^1^Department of Internal Medicine, William Beaumont Hospital, Royal Oak, MI 48073, USA; ^2^Department of Gastroenterology, William Beaumont Hospital, Royal Oak, MI 48073, USA; ^3^Oakland University William Beaumont School of Medicine, Royal Oak, MI 48073, USA

## Abstract

A 59-year-old man with past medical history of thoracic aortic aneurysm treated with thoracic endovascular aortic repair presented with melena for 2 weeks. Initial EGD did not reveal the source of bleeding and showed normal esophagus; abdominal arteriogram did not reveal a fistulous communication and initial CTA showed normal position of the aortic graft stent without endoleak. The sixth EGD revealed a submucosal tumor-like projection in the upper esophagus and stigmata of recent bleeding. Another thoracic endovascular aortic repair with stent was placed over the old graft for presumed aortoesophageal fistula. Poststent upper gastrointestinal series with contrast showed extravasation of the contrast from the esophagus and CTA showed fistulous tract between aorta and esophagus. The patient refused definitive surgical repair despite having infected aortic graft; jejunostomy tube was placed and life-long suppressive antibiotic treatment was given and the patient is doing well at 2-year follow-up.

## 1. Introduction

Thoracic endovascular aortic repair (TEVAR) has become one of the main therapies for thoracoabdominal aortic diseases including thoracic aortic aneurysm (TAA), traumatic aortic transection, or aortoesophageal fistula (AEF). Although TEVAR is preferred over open surgical repairs for AEF treatment due to better perioperative morbidity and mortality, it is associated with high rate of reintervention due to its complications that occur after TEVAR. TEVAR is performed more frequently despite the lack of randomized controlled trial proving the long-term effectiveness of TEVAR over open surgical repair. AEF is one of the catastrophic complications of TEVAR, although TEVAR itself can be used as a temporary treatment for AEF. In this case, we are presenting AEF that developed as an unusual late complication after an initial TEVAR to treat traumatic thoracic aortic aneurysm; this AEF was treated with another TEVAR and the patient is doing well at 2-year follow-up despite infected old aortic graft that was not removed.

## 2. Case Presentation

A 59-year-old man with past medical history of repaired saccular descending TAA 5 years ago presented with melanotic stools, progressively worsening epigastric discomfort, low-grade fever, and fatigue for 2 weeks with no episodes of nausea, vomiting, or hematemesis. The patient was taking at least 6 tablets of full-dose aspirin for joint pain daily. Colonoscopy revealed old blood in terminal ileum and esophagogastroduodenoscopy (EGD) revealed gastric petechiae and hiatal hernia. He received proton pump inhibitor for hemorrhagic gastritis and quit taking NSAIDs. After 2 weeks, he presented to our facility with recurrent episodes of melena only without hematemesis. Physical examination revealed stable vital signs, pallor, and soft, nontender abdomen. Laboratory analysis showed hemoglobin = 6.7 g/dL with normal platelet count and normal coagulation profile. Serum parameters of liver and kidney functions were normal. An emergent EGD revealed large adherent blood clot along the greater curvature of the stomach, which could not be removed, and there was no evidence of active bleeding. The patient was started on proton pump inhibitor continuous infusion for upper gastrointestinal (GI) bleeding from the stomach. Computerized tomography with angiography (CTA) revealed an unchanged position of the aortic graft stent without endoleak.

## 3. Hospital Course

**Figure 1:**
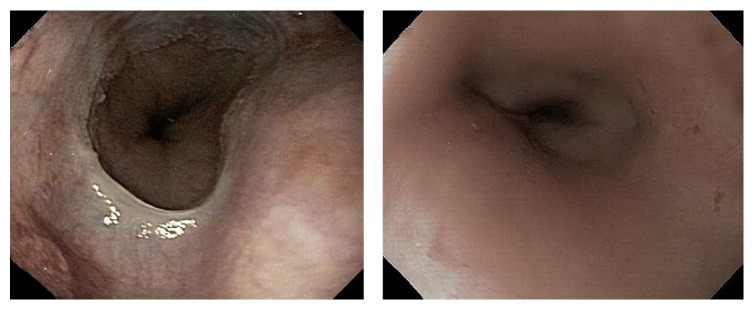
Initial EGDs showed normal esophagus with no evidence of aortoesophageal fistula. No evidence of bleeding in the esophagus.

**Figure 2:**
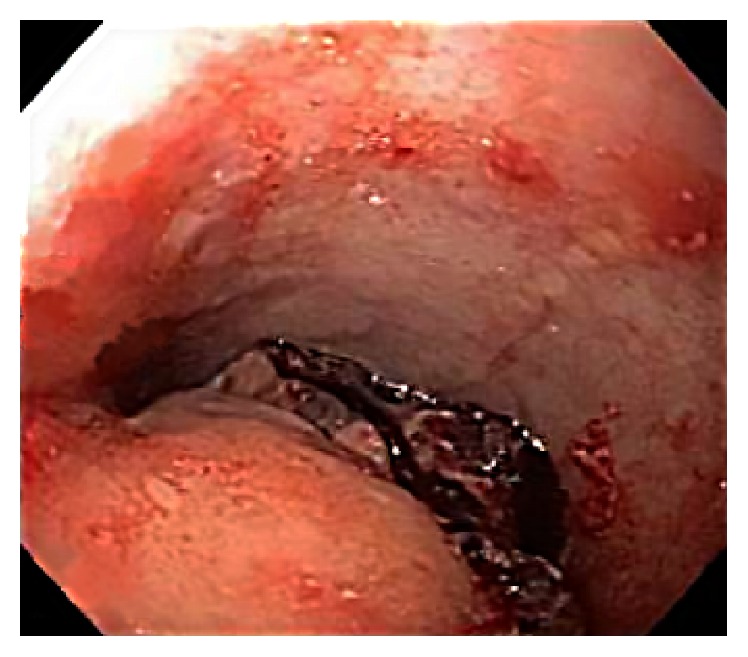
Esophagogastroduodenoscopy (EGD) showing submucosal tumor-like projection secondary to extrinsic compression of the upper esophagus with blood clot sitting on top of the compression at 5 cm below the upper esophageal sphincter (UES).

**Figure 3:**
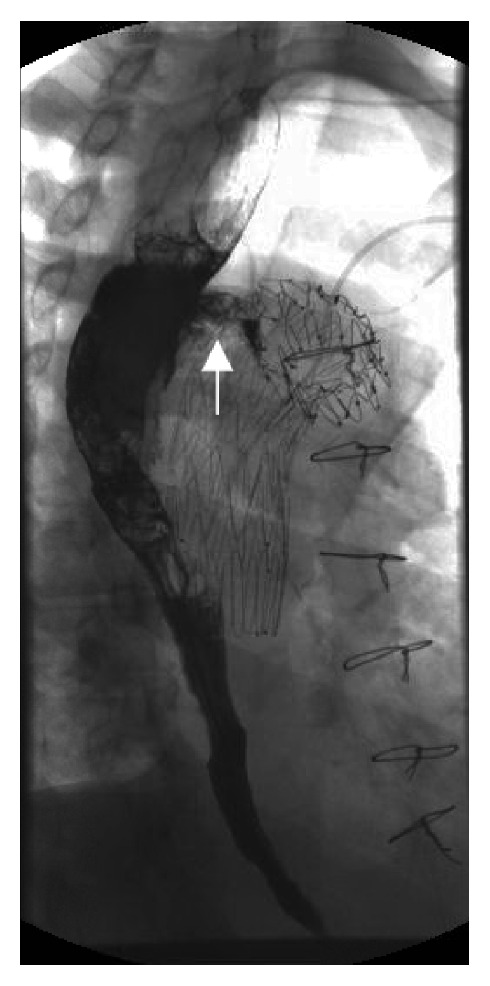
Upper gastrointestinal study with barium contrast showing extraluminal contrast seen arising from proximal esophagus extending outward visualizing the fistulous tract.

**Figure 4:**
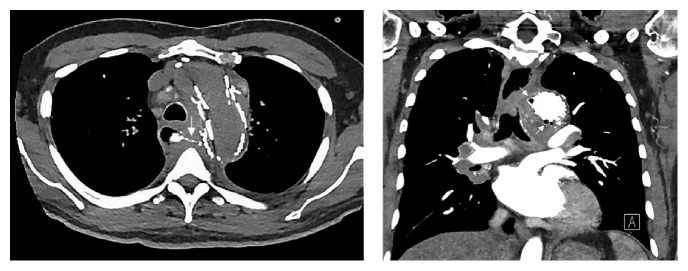
CT angiography axial view showing linear high-density contrast collection extending from esophageal wall towards the aortic graft representing retained barium contrast from the patient's recent upper gastrointestinal series visualizing fistulous tract and the coronal view showing multiple foci of gas or air bubbles adjacent to the patient's aortic stent graft.

**Figure 5:**
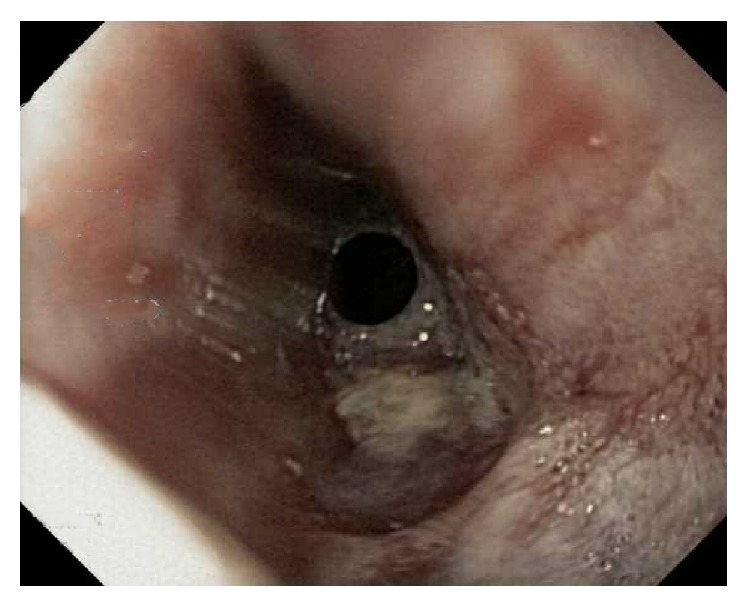
Follow-up EGD showed granulation tissue with inflammation at the site of fistula but no bleeding, no pulsating mass, and no ulcer.

A follow-up EGD showed more extensive blood clot adherent to the same location that could not be removed. Despite commencement of proton pump inhibitor infusion, the patient continued to have melanotic stool. Abdominal arteriogram revealed no contrast extravasation from the blood vessels, and empiric embolization of the left gastric artery was performed as the source of bleeding was thought to be from a lesion underlying the blood clot along the greater curvature of the stomach. Over the next few days, he continued to have melena and he required transfusion of 12 units of packed red blood cells. Multiple EGDs (Figure 1) were performed, which showed stigmata of recent bleeding with normal esophagus. He developed fever spikes, blood cultures were positive for* Streptococcus* viridans, and the appropriate antibiotics were given. Extensive diagnostic tests were performed including colonoscopy; Technetium-Labeled Red Blood Cell Bleeding Scan and CTA of the abdomen were insignificant. The sixth EGD revealed coagulated blood in the upper one-third of the esophagus overlying a tumor-like submucosal projection secondary to extrinsic compression 5 cm below the upper esophageal sphincter (Figure 2), which was not seen on previous EGDs. TEVAR was performed under fluoroscopic guidance for presumed AEF and a new stent graft was placed over the previous graft with resolution of the GI bleeding. Upper gastrointestinal series (UGIS) with barium revealed extraluminal contrast arising from the esophagus at the level of the aortic arch (Figure 3). A repeat CTA revealed linear high-density tract extending from the left esophageal wall towards the aorta as well as multiple air bubbles adjacent to the aortic graft (Figure 4). These findings confirmed the presence of AEF with closure of aortic side by the recently placed stent. The patient refused to have a definitive open surgical repair. Jejunostomy tube was placed, and life-long suppressive antibiotic treatment is being given as the infected graft is still in place. Follow-up EGD (Figure 5) showed granulation tissue with surrounding inflammation around the fistulous opening with no evidence of recent bleeding and follow-up UGIS showed extravasation of the contrast at the site of the fistula without spillage of the contrast into the aorta. The patient is doing well at 2-year follow-up.

## 4. Discussion

AEF is a condition that is defined as an abnormal communication between the esophagus and the aorta [1]. AEF occurs primarily due to local disease in the aorta or esophagus including aneurysms, malignancies, and ulcers or secondary to other factors that can cause damage to these organs including presence of foreign bodies, prosthetic grafts, and stents [2, 3]. Hollander and Quick [1] evaluated 500 patients with AEF, revealing that the most common cause of AEF is TAA (256 cases out of 500; 54.2%), followed by foreign body ingestion (93/500; 19.2%) and esophageal malignancy (85/500; 17%). Prosthetic grafts represented a small number of cases (7/500; 1.4%) with no clear definition whether stents were included or not.

The number of patients undergoing TEVAR has increased significantly in the last 2 decades due to the minimally invasive nature of this technique. AEF is a potential complication of TEVAR that usually occurs within 1–16 months after the procedure and is invariably fatal [4]. Eggebrecht et al.'s [4] study revealed that 6 patients out of 268 developed AEF as a complication of TEVAR (incidence 1.9%). Different mechanisms of AEF development following TEVAR have been postulated including direct erosion of the stent graft into the esophagus, pressure necrosis of the self-expanding prosthesis, ischemic necrosis of the esophagus due to interruption of its vasculature, infection of the stent graft, and the presence of an endoleak [5, 6]. AEF usually occurs within few months after TEVAR, but Kouritas et al. [7] reported one case of AEF that developed 6 years after TEVAR.

Hollander and Quick [1] mentioned in their study that 63% of the patients had sentinel hematemesis, 58% had chest pain, and 42% had dysphagia. Chiari's triad of chest pain and sentinel hematemesis followed by massive hematemesis has been reported in the literature as a common characteristic presentation in patients with AEF [6, 8–12]. Other less frequent presentations include mediastinitis or fever [4, 10]. One case of AEF with positive blood cultures for* Streptococcus* viridans has been reported [3] and this case is the second one. Complications of AEF include mediastinitis, infection of the surgical or endovascular graft, sepsis, and death. Thus, AEF can be a cause or can develop as a complication of graft infection [3].

EGD is the most sensitive and specific modality for diagnosis of AEF [6, 8, 9, 13] and it is the first test used for diagnosis although its sensitivity for detecting AEF is 38% [14]. The most common finding of AEF on EGD is submucosal tumor-like protrusion; however, extrinsic compression, ulcerative lesion, pulsating protrusion with central fistula, and exposure of the aortic stent have been reported as other findings on EGD [11]. CTA might show an extravasation of the contrast material outside the aorta, which is considered a definite sign; however, it is rarely seen [3, 6, 15]. We should suspect AEF when we see the following findings on a CT following TEVAR: mediastinal air, aortic lumen air, persistent or expanding perigraft fluid, soft tissue density beyond 6 months from the date of the procedure, or loss of the aortoesophageal fat plane. An UGIS is an easy way to diagnose AEF as it can visualize an extravasated contrast arising from the esophageal side of the fistula, which is also considered a definite sign [2].

Development of AEF has a very poor prognosis [16] and the condition is generally fatal. The only definitive treatment of AEF is surgical repair and it has to be initiated as soon as possible [8, 9], definitive surgical management includes left thoracotomy with aortic graft replacement, esophageal wall repair, and removal of the fistula, and antibiotics are used as an adjunct to surgery [3]. Using stent graft placement for management of AEF is controversial as there is a theoretical increase in the risk of endoleak after stent placement due to weakening of the aortic wall that already exists by inflammatory changes associated with AEF development [3]. Insertion and inflation of a Blakemore tube can be used to stop bleeding temporarily until patients undergo surgery [17–19]. Xi et al. [20] revealed the advantages of TEVAR over open surgical repair for AEF, which are as follows: it is well tolerated by high risk patients, minimizes risk of cardiovascular disease, reduces mortality, can be performed under sedation and local anesthesia, and is a rapid method to minimize blood loss even in unstable patients, but some studies [3, 20, 21] also suggested that TEVAR should be used as a stop gap measure before definitive surgical repair. Burks Jr. et al. [22] revealed in their case series that TEVAR is a safe method for bleeding control, when it is combined with long-term antibiotic treatment and percutaneous drainage if there is fluid collection or may be combined with esophageal diversion proximal to AEF. Many patients died of coexistent cardiopulmonary disease before infection or aortic degeneration becomes a significant problem; however, it is not a definitive therapy and complete eradication of infection is impossible without debridement of all contaminated prosthetic infected tissues. Antibiotics alone are not recommended as a primary measure to treat AEF because antibiotics alone will not eliminate infection in an infected foreign body [3].

This case had an atypical presentation; he did not present with Chiari's triad. He developed symptoms after 5 years of TEVAR, he underwent 5 EGDs, which showed blood in the stomach without findings suggestive of AEF especially in the setting of a normal esophagus, and the source of bleeding could not be identified until the characteristic tumor-like submucosal protrusion was noticed on the sixth EGD. The normal abdominal arteriogram might be due to the exclusion of the thoracic part of the aorta from the study. Also the initial CTA did not show extravasation of the contrast or other findings suggestive of AEF. The combination of the history of aortic stent graft, upper GI bleeding and endoscopic findings on the sixth EGD, raised the suspicion for AEF and we proceeded with another TEVAR for a presumed AEF and the patient improved after the procedure. UGIS was performed after the second TEVAR, which revealed extraluminal contrast confirming the presumed diagnosis of AEF and the contrast used in UGIS was visualized in the CTA that was performed after TEVAR; this contrast is related to the barium contrast used in UGIS and not the injected contrast of the CTA as the aortic side of the AEF is blocked by the recently placed stent. The patient refused open surgical repair as his symptoms improved after TEVAR, so jejunostomy tube was necessary to avoid continuous food passage into the fistula, which may lead to mediastinitis. Life-long suppressive antibiotics were the only option available although it is not recommended to leave an infected stent without removal especially in the setting of a 59-year-old man who is not old and without comorbid conditions as this will affect his quality of life significantly, but he refused any surgical repairs at least for the meantime. The patient tried to eat soft food, but he developed fever, so he was advised not to swallow anything until definitive surgical repair is performed as it may cause life-threatening mediastinitis. We believe the cause of late AEF formation is either due to delayed infection of the stent or an atypical slow inflammatory changes developed over years. The management was different in this case as the patient refused the recommended definitive treatment. We are presenting this case to share the complicated atypical presentation; despite having negative imaging, the diagnosis of AEF was not ruled out. Diagnosis was confirmed retrospectively after the second TEVAR was performed with UGIS and CTA.
